# Determinants of health status in older patients with transthyretin cardiac amyloidosis: a prospective cohort study

**DOI:** 10.1007/s40520-024-02750-6

**Published:** 2024-04-10

**Authors:** Carlo Fumagalli, Lucia Ponti, Martina Smorti, Francesca Pozza, Alessia Argirò, Mattia Zampieri, Carlo Di Mario, Raffaele Marfella, Celestino Sardu, Giuseppe Paolisso, Iacopo Olivotto, Federico Perfetto, Andrea Ungar, Niccolò Marchionni, Francesco Cappelli

**Affiliations:** 1 Department of Advanced Medical and Surgical Sciences, University of Campania “Luigi Vanvitelli”, Naples, Italy; 2 Cardiomyopathy Unit, Careggi University Hospital, Florence, Italy; 3 Tuscan Regional Amyloidosis Centre, Careggi University Hospital, Florence, Italy; 4 Department of Humanities, University of Urbino, Urbino, Italy; 5 Department of Surgical, Medical and Molecular Pathology and Critical Care Medicine, University of Pisa, Pisa, Italy; 6 IV Internal Medicine Division, Careggi University Hospital, Florence, Italy; 7 Division of Interventional Structural Cardiology, Cardiothoracovascular Department, Careggi University Hospital, Florence, Italy; 8 Department of Experimental and Clinical Medicine, University of Florence, Florence, Italy; 9 Meyer Children Hospital, Florence, Italy; 10 Geriatric Intensive Care Unit, University of Florence, Florence, Italy

**Keywords:** ATTR-CA, Frailty, Social environment, Quality of life, KCCQ

## Abstract

**Background:**

Whether, and to what extent, frailty and other geriatric domains are linked to health status in patients with transthyretin cardiac amyloidosis (ATTR-CA) is unknown.

**Aims:**

To determine the association of frailty with health status [defined by the Kansas City Cardiomyopathy Questionnaire (KCCQ)] in patients with ATTR-CA.

**Methods:**

Consecutive ATTR-CA patients undergoing cardiovascular assessment at a tertiary care clinic from September 2021 to September 2023 were invited to participate. KCCQ, frailty and social environment were recorded. Frailty was assessed using the modified Frailty Index (mFI), mapping 11 variables from the Canadian Study of Health and Aging (frailty ≥0.36).

**Results:**

Of 168 screened ATTR-CA patients, 138 [83% men, median age of 79 (75–84) years] were enrolled in the study. Median KCCQ was 66 (50–75). wtATTR-CA was the most prevalent form (N = 113, 81.9%). The most frequent cardiac variant was Ile68Leu (17/25 individuals with vATTR-CA). Twenty (14.5%) patients were considered frail, and prevalence of overt disability was 6.5%. At multivariable linear regression analysis, factors associated with worsening KCCQ were age at evaluation, the mFI, NYHA Class, and NAC Score. Gender, ATTR-CA type, phenotype, and LVEF were not associated with health status.

**Discussion:**

In older patients diagnosed with ATTR-CA, frailty, symptoms, and disease severity were associated with KCCQ.

**Conclusions:**

Functional status is a determinant of quality of life and health status in older individuals with a main diagnosis of ATTR-CA. Future research may provide more in-depth knowledge on the association of frailty in patients with ATTR-CA with respect to quality of life and prognosis.

**Supplementary Information:**

The online version contains supplementary material available at 10.1007/s40520-024-02750-6.

## Introduction

Transthyretin cardiac amyloidosis (ATTR-CA) is a condition characterized by the abnormal deposition of misfolded transthyretin in the heart. There are two types of ATTR: a rare, inherited genetic variant of TTR (vATTR) and a more common, non-inheritable wild type TTR (wtATTR) [1]. In the last decade, referral centers for ATTR-CA have witnessed a profound change in the epidemiology of ATTR-CA, whereby patients are diagnosed at an older age, with a higher burden of comorbidities translating into greater complexity but with less advanced cardiac amyloid involvement as evidenced by higher percentage of National Amyloid Center (NAC) staging system stage I and II compared to the past [2]. While early diagnosis is crucial to improve outcome [3, 4], particularly after the emergence of new disease-modifying drugs, individual patient examination has shifted from a limited instrumental evaluation to a broader assessment including the definition of functional capacity and, most importantly, health status/quality of life [typically assessed with the Kansas City Cardiomyopathy Questionnaire (KCCQ) [1]]. These tools, albeit informative, may not reflect the clinical complexity of older patients with ATTR-CA. For instance, recent data showed not only that depression and anxiety are extremely frequent among ATTR-CA patients, but also how they can influence the way in which patients perceive and report their cardiac symptoms [5, 6]. Given the high prevalence of patients diagnosed at 80+ years [7], assessment of frailty (defined as a decline in functioning across multiple physiological systems and an increased vulnerability to stressors [8]) may prove useful within a more comprehensive evaluation to better describe individuals candidate to novel therapies and identify critical issues to improve healthcare: chronic conditions and frailty in the aging population are associated with reduced care effectiveness and impaired quality of life, which negatively affect long-term outcome [8–10]. Whether, and to what extent frailty and other factors stemming from a comprehensive geriatric assessment may be associated with health status in patients with ATTR-CA, has never been explored.

We aimed to assess the association of frailty with health status (as expressed with the KCCQ), in a prospective cohort of older ATTR-CA patients followed in the referral center for cardiac amyloidosis management.

## Methods

### Participants and procedures

All consecutive patients diagnosed with ATTR-CA undergoing routine cardiological evaluations at a high-volume regional Referral Amyloidosis Centre between September 2021 to September 2023 were invited to participate in a prospective cohort study aimed at assessing quality of life and health status. The local Ethics Committee approved the study (CEAVC; protocol number 19476_OSS/2021). The study protocol has been previously described: a part of the present prospective study cohort was previously included in another analysis aimed at evaluating the burden of social factors in patients diagnosed with ATTR-CA [6]. Participation was voluntary and, before starting data collection, written informed consent was recorded for all participants. Patients with diagnosed cognitive impairment and inability to understand Italian were excluded. The sample size of the study was determined from an a priori statistical power analysis using the G-Power 3.1 software. For a statistical power of 95%, an effect size of 0.15, an alpha at 0.05, and up to 7 predictors, the necessary sample size was 89.

### Frailty evaluation and social environment

Frailty was assessed using the modified Frailty Index (mFI), mapping 11 variables (non-independent functional status, history of diabetes mellitus, chronic obstructive pulmonary disease or pneumonia, heart failure, myocardial infarction, angina or coronary revascularization, hypertension, peripheral vascular disease, presence of impaired sensorium, TIA or cerebrovascular event without or with deficit) from the 70-item Canadian Study of Health and Aging Frailty Index and validated in other clinical settings [11, 12], which was recently recommended together with the Frail Phenotype by the ‘Frailty in Cardiology Consensus document’ by the European Society of Cardiology [13]. Frailty was defined by a ratio of actual over total conditions ≥0.36. All patients were also interviewed for social environment: in particular, information regarding marital status and cohabitant companions (‘alone’ or ‘not alone’) was obtained for each candidate. To better describe clinical characteristics by patients’ social environment, the cohort was also described by patients living alone or not.

### Health status and symptoms perception

The subjective perception of symptoms severity was measured using the Italian version of the Kansas City Cardiomyopathy Questionnaire (KCCQ) [14]. The KCCQ is an instrument commonly used to assess the perceived burden of HF symptoms and health status through 23 items. The total score ranges from 0 to 100, with higher scores meaning better health perception.

### Clinical evaluation

Cardiological assessment, the type of ATTR, months since diagnosis, New York Heart Association (NHYA) class, and National Amyloid Center (NAC) staging system [15] were systematically recorded, in a standardized manner. Disease duration was defined as the time interval from first diagnosis and study interview. Resting NT-proBNP plasma levels (ECLIA Roche) and glomerular filtration rate (GFR) according to Modification of Diet in Renal Disease formulas were also measured in a time window of 2 weeks from the questionnaire evaluation. The NAC staging system was derived by comparing values of GFR and NT-proBNP [15]. Echocardiographic variables included: left ventricular (LV) posterior wall (PW) and interventricular septum (IVS) thickness, LV end diastolic diameter (LVEDD), left atrium diastolic diameter (LADD), LV ejection fraction (LVEF), and the ratio between early diastolic transmitral flow and early diastolic mitral annular motion (E/e’) to assess diastolic dysfunction. All patients followed in our Centre were offered genetic test as part of the routine clinical assessment. Genetic status (v- vs wt-ATTRCA) and mutation type were acquired.

### Primary endpoint

To describe the association of frailty (expressed as mFI) with the KCCQ.

### Statistical analysis

Continuous variables are presented as median and interquartile range (IQR). Categorical variables are presented as counts and percentages. To better describe clinical characteristics by patients’ social environment, the cohort was also described by frailty status. A multivariable linear regression model adjusted for overlap effects, with stepwise backward deletion of redundant variables, was used to study candidate factors potentially associated with KCCQ with a *p* < 0.10 at univariable analysis. The model was built by adjusting first for cardiac variables which are typically associated with KCCQ in ATTR-CA (first block) and later for the mFI and social variables after a preliminary univariable analysis (*p* for inclusion <0.10) A final two-sided *p *value <0.05 was considered as statistically significant. All analyses were performed using IBM SPSS Statistics for Macintosh, Version 28.0 (Armonk, NY: IBM Corp., USA). Graphs were generated with GraphPad Prism v. 10.2.2. The Graphical Abstract was designed via BioRender.com.

## Results

### Prevalence of frailty in ATTR-CA

Over a period of 24 months, a total of 168 consecutive patients were invited. Of them, 10 (5.9%) refused to participate because of reported lack of time to complete the questionnaires and 9 (5.3%) were positive screening for cognitive impairment. Eleven (6.5%) were excluded because of incomplete data. The demographic and clinical characteristics are presented in Table 1. The final sample consisted of 138 patients (115 men, 83%) with a median age of 79 (75–84) years. wtATTR-CA was the most prevalent form (N = 113, 81.9%). The most frequent cardiac variant was Ile68Leu (17/25 individuals with vATTR-CA). Most patients were in NYHA Class I or II, with >50% being in Stage I according to the NAC score. Median KCCQ was 66 (50–75). Only 2 patients had received Tafamidis for >12 months before evaluation.

**Table 1:** Demographic and clinical characteristics of the study population

	Overall population N = 138	Non-frail N = 118	Frail N = 20	*p* value
*Demographic characteristics*				
Age, median (IQR) (years)	79 (75–84)	79 (75–84)	84 (78–87)	0.028
Men, N (%)	115 (83.3)	99 (83.9)	16 (80.0)	0.665
ATTR type				
WT-, N (%)	113 (81.9)	99 (81.4)	14 (70.0)	0.204
V-, N (%)	25 (18.1)	19 (16.1)	6 (30.0)	
Ile68Leu, N (%)	17/25	13	4	
Val122Ile, N (%)	3/25	2	1	
Glu74Gln, N (%)	1/25	1	0	
Phe84Ile, N (%)	1/25	1	0	
Val30Met, N (%)	1/25	1	0	
Mixed Phenotype, N (%)	3 (2.2)	1 (0.8)	2 (10.0)	0.055
KCCQ, median (IQR) (score)	66 (50–75)	69 (54–80)	48 (37–57)	<0.001
Disease duration, median (IQR) (months)	23 (10–42)	23 (11–36)	35 (18–47)	0.009
NYHA Class III/IV, N (%)	31 (22.5)	19 (16.1)	12 (60.0)	<0.001
NAC Score				
Class I, N (%)	80 (57.9)	75 (63.6)	5 (25.0)	0.004
Class II, N (%)	42 (30.4)	31 (26.2)	11 (55.0)	
Class III, N (%)	16 (11.5)	12 (10.2)	4 (20.0)	
Modified Frailty Index—11 items, median (IQR)	0.09 (0–0.36)	–	–	
≥0.36, N (%)	20 (14.5)	–	–	
Ischemic Heart Disease, N (%)	16 (11.5)	11 (9.3)	5 (20.0)	0.058
Type 2 Diabetes Mellitus, N (%)	23 (16.7)	16 (13.6)	7 (35.0)	0.017
Stroke/TIA, N (%)	13 (9.4)	9 (7.6)	4 (20.0)	0.078
Social Disability, N (%)	9 (6.5)	2 (1.7)	7 (35.0)	<0.001
Widow, N (%)	25 (18.1)	20 (16.9)	5 (20.0)	0.387
Living alone, N (%)	28 (20.3)	24 (17.4)	4 (20.0)	0.972
*Instrumental Evaluation*				
Left Atrial Diameter, median (IQR) (mm)	45 (41–50)	45 (41–50)	46 (40–51)	0.478
Interventricular septal thickness, median (IQR) (mm)	16 (15–18)	17 (16–20)	17 (10, 16–20)	0.126
Posterior Wall, median (IQR) (mm)	15 (14–15)	15 (14–17)	15 (14–18)	0.261
LVEDD, median (IQR) (mm)	44 (40–48)	44 (40–48)	43 (39–46)	0.902
LVEF, median (IQR) (%)	57 (50–61)	58 (50–61)	55 (45–60)	0.083
LVEF < 50%, N (%)	28 (20.3)	21 (17.8)	7 (35.0)	0.077
E/e’, median (IQR) (%)	15 (12–19)	14 (11–17)	18 (14–20)	0.098

*ATTR* transthyretin cardiac amyloidosis, *NAC* National Amyloidosis Centre, *IQR* Interquartile Range, *LVEDD* Left Ventricular End Diastolic Diameter, *LVEF* Left Ventricular Ejection Fraction, *NYHA* New York Heart Association, *TIA* Transient Ischemic Attack, *V* variant, *WT* wild type

Overall, 20 (14.5%) patients were considered frail according to the mFI and prevalence of social disability was 6.5%. At echocardiographic evaluation (Table 1), median interventricular septal thickness was 16 (15–18) mm. The prevalence of systolic dysfunction was 20.3%. Overall, 3 patients presented with a mixed phenotype, with a trend towards a higher prevalence among frail individuals.

Social environment analysis revealed that 25 (18.1%) patients were widowers and 28 (20.3%) were living alone. The main clinical characteristics by frailty status are summarized in Table 1. Frail patients were older at evaluation, with a longer history of the disease, higher prevalence of NAC III stage, worse KCCQ scores and higher prevalence of diabetes. However, in terms of echocardiographic evaluation, no meaningful differences were captured.

### Factors associated with perceived quality of life

**Fig. 1:**
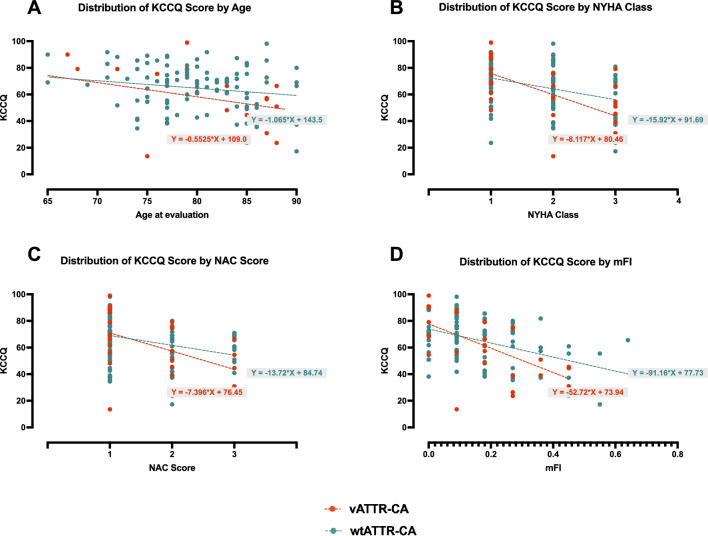
Distribution of the Kansas City Cardiomyopathy Questionnaire (KCCQ) by age, NYHA Class, NAC Score, and levels of modified frailty index-11 items (mFI)

Perceived quality of life assessed with KCCQ deteriorated with increasing age, NYHA Class, NAC stage and worsening levels of mFI (and thus frailty), in patients with both wtATTR-CA and vATTR-CA (Fig. 1).

Univariable and multivariable regression analyses presented in Table 2. At multivariable linear regression analysis, age at evaluation, the mFI, NYHA Class and the NAC score were associated with significant changes in KCCQ. By contracts, gender, ATTR-CA type (wt- vs v- CA), phenotype, LVEF and ability of living alone were excluded from the model at univariable analysis (Table 2).

**Table 2:** Factors associated with KCCQ at multivariable linear regression analysis

	Univariable						Multivariable					
	Coefficients		t	*p*	95.0% CI β		Coefficients		t	*p*	95.0% CI β	
	β	SE			Lower	Upper	β	SE			Lower	Upper
Age	−0.738	0.189	−3.96	<0.001	−1.101	−0.369	−0.355	0.176	−2.02	0.046	−0.702	−0.007
Disease duration (per Δ month)	−0.174	0.063	−2.72	0.007	−0.301	−0.047	−0.052	0.059	−0.88	0.381	−0.169	0.065
mFI (per Δ increase)	−60.640	9.885	−6.13	<0.001	−80.198	−41.082	−39.184	10.752	−3.64	<0.001	−60.462	−17.900
NYHA class (per class increase)	−17.229	3.492	−4.93	<0.001	−24.140	−10.319	−7.619	3.590	−2.12	0.036	−14.726	−0.511
NAC Score (per Δ class)	−8.872	2.142	−4.14	<0.001	−13.111	−4.663	−4.389	2.106	−2.08	0.039	−8.589	−0.220
Phenotype (pure cardiac vs mixed)	2.133	0.740	1.27	0.792	−1.689	7.940						
Male Gender	−2.980	4.284	−0.70	0.488	−11.457	5.490						
LVEF	24.497	16.159	1.52	0.132	−7.468	56.463						
Alone	5.410	4.856	1.11	0.267	−4.195	15.016						
Widow	−5.836	4.050	−1.44	0.152	−18.847	2.175						

*CI* confidence intervals, *LVEF* left ventricular ejection fraction, *mFI* modified frailty index, *NAC* National Amyloidosis Centre, *NYHA* New York Heart Association Class, *SE* standard error

## Discussion

In the present prospective analysis of older patients diagnosed with ATTR-CA we found that: (1) prevalence of frailty, defined by the mFI, among patients actively invited for an interview on quality of life, was 14.5%; (2) higher levels of the modified Frailty Index were associated with worse perceived quality of life and health status for patients with both wt- and v-ATTR-CA; (3) at multivariable linear regression analysis, age at evaluation, the modified Frailty Index, NYHA class, and NAC stage were associated with worse perceived quality of life, while ATTR-CA type, phenotype, and LVEF were not.

The non-negligible prevalence of frailty among patients with ATTR-CA (>1-in-7 patients) who are considered cognitively eligible for health status assessment suggests that a comprehensive and interdisciplinary assessment may be useful once a diagnosis is established. It’s crucial to note that data on frailty in ATTR-CA patients remain limited and its determination is an active area of investigation. Recent evidence suggests that the prevalence of frailty among these patients is highly variable and potentially higher than what has been measured (from 15 to 60%) [16–18], and may be linked to worse perceived social support [17]. There are multiple reasons that might explain such high variability in the literature: for one thing different tools adopted to assess frailty (either the Clinical Frailty Scale, Short Emergency Geriatric Assessment, or the mFI-11 items) may yield a different prevalence depending on the factors that are being considered. Furthermore, the time span of patient recruitment may play a major role. In the report by Nowell Fine and collaborators [16, 18], patients were enrolled over a period of 5 years, from 2014 to 2019, with ~60% showing at least a mild degree of frailty (CFS > 4). A lower prevalence was noted in another report of patients enrolled within 1 year [18]. A selection bias of the *fittest* due to study enrollment criteria could also be present—patients with cognitive decline were excluded by study protocol, as this may have tampered with KCCQ analysis. Although dementia and frailty are two separate geriatric entities, we acknowledge that this might have led to a selection bias with only patients on the lower spectrum of the mFI being included. Notably, in our cohort, prevalence of NYHA I/II was high, but in line with recent evidence which suggest a shift towards milder ATTR-CA phenotypes in the last years, with higher percentage of NAC stage I^2^. Different frailty prevalence has been frequently observed in patients with HF and valvular heart disease assessed for geriatric syndromes and should come to no surprise, given the high heterogeneity of clinical and functional phenotypes of older patients undergoing a CGA [19, 20]. Overall, older patients with ATTR-CA may benefit from a CGA. Recognition of geriatric syndromes (such as frailty, functional decline, disability, malnutrition, etc.) could allow for earlier personalized care, improving quality of life and outcomes. Although the CGA is a diagnostic process, it may also be used as a tool to monitor patients in the long term. Furthermore, frailty assessment tools or the CGA may help identify patients who could benefit from disease-modifying therapy, as opposed to those who are at high risk of futility or potential harm from treatment [21].

In our cohort, median values for KCCQ were 66 (50–75) points, in line with other recent observational studies [22, 23]. Until recently, ATTR-CA was considered a progressive disease characterized by poor and rapidly worsening quality of life [22]: advanced age, diagnostic delay, systemic symptoms, and multiple comorbidities are all factors which may potentially account for this trend. Recently, data from the Nordic PROACT study, a multicenter cross-sectional non-interventional study, confirmed this concept and suggested a direct association of severity of heart failure symptoms (i.e. NYHA Class) with worsening levels of patient reported outcomes (PROMS) such as KCCQ and European Quality of Life 5 Dimensions 5 Level Version (EQ-5D-5L) [24]; interestingly, other variables, including instrumental assessment, were not correlated. These results confirm that in older patients diagnosed with heart failure (with either reduced or preserved ejection fraction), NYHA class is a strong marker of quality of life and any change in reported symptoms should be promptly investigated [25, 26]. Our findings, although partly consistent with existing literature, significantly enhance the evaluation of PROMS in ATTR-CA by a new geriatric domain which is now gathering momentum, as it encompasses functional, sensory, and clinical dimensions. They also indicate that traditional cardiological assessments in this context may offer limited discriminatory capacity in describing the KCCQ and, more broadly, the quality of life. With aging, determinants of quality of life in patients diagnosed with heart failure may depend more upon functional limitation (progressing to overt disability), fatigue, and social environment [27]: given the close link of quality of life to “hard” outcomes such hospitalization or mortality [28], patients should be routinely assessed for symptoms and multidomain impairment. On a broader note, given the promising impact of new disease modifying drugs on KCCQ [29], it may be speculated that the CGA and frailty could be used as surrogate markers for disease ATTR-CA trajectories and consulting geriatricians could provide useful insight into functional status and disability [30].

In our study, prevalence of overt disability was low; however, higher levels of KCCQ (suggesting better quality of life) in patients capable of successfully living alone might suggest that health status could be a parameter which can be modulated by disability or dependency (or lack thereof).

In general, health status is a complex entity to assess, especially in the aged. In our cohort, three patients with low mFI-11 (i.e. not frail) presented with poor KCCQ values: in two cases, patients either had an advanced disease stage with a history of congestive heart failure (NYHA Class III during evaluation) and had suffered a disabling stroke or had suffered a major stroke which produced functional disability with higher degree of dependency (and inability to live alone). A third patient with an mFI of 0.09 was recovering from a period of major depression following the death of the next of kin.

Although these patients can be considered outliers when considering mFI alone, they also confirm that KCCQ, and more broadly, quality of life in older patients, is the result of multiple key components (e.g. comorbidities, functional status, dependency, depression etc [5]).

### Limitations

Our study has some limitations: first, its transversal design, preventing more accurate and informative longitudinal analysis of the associations of frailty, structure of social environment, and quality of life with incident outcomes. We assessed frailty using the mFI. Two main models have been proposed to define frailty: the *phenotypic* and the *multiple-deficits model*. The first one, designed by Fried and colleagues [31], identifies frailty by the presence of more than two of five features: (a) unintentional weight loss; (b) rapid exhaustion; (c) low physical activity level; (d) slow walking speed; (e) muscle weakness. The second one rates frailty by the number of functional, sensory and clinical deficits based on the Canadian Study of Health and Aging Frailty (overall 70 items) [12], which contains the 11 variables used to calculate the mFI [11]. Following these two models, other scale were developed, like the Edmonton Frailty Scale, which have been assessed also in HF and heart transplantation [32]. We must acknowledge that the mFI adopted in our study does not measure several factors, such as laboratory or motor parameters, which may have been linked to frailty and could have helped achieve a better patient description. Furthermore, we acknowledge that the mFI-11 may not give the possibility to identify different degrees of frailty. A more comprehensive geriatric assessment in these patients might have had the potential to better identify frailty and disability. While we cannot exclude a potential impact of a mixed phenotype on frailty assessment and quality of life, the present population is likely underpowered to identify any meaningful association.

Finally, the role of disease modifying drugs (e.g. Tafamidis) was not explored in the present paper: our study terminated patient enrolling in September 2023. In Tuscany, Tafamidis prescription started as of January 2022. Referral to prescription most of the times followed frailty assessment. Since patients are evaluated every 6 months by our Center protocol, no patient with Tafamidis was able to complete the first follow up visit. By September 2022, only two patients had received Tafamidis for a considerable amount of time (>12 months). Given the limited number of patients and restricted exposure to the drug to produce tangible effects on KCCQ based on data from the ATTRACT trial, we did not include this in the multivariable model [23].

## Conclusions

In conclusion, in older patients diagnosed with ATTR-CA, age, frailty, NYHA Class and disease stage were associated with KCCQ, while systolic function was not. These results reinforce the concept that overall functional status is a determinant of quality of life and health status in older individuals with a main diagnosis of ATTR-CA. Future research may provide more in-depth knowledge on the association of frailty with quality of life in patients with ATTR-CA and, most importantly, to assess whether—and to what extent—frailty is an independent determinant of prognosis, to be considered in therapeutic decision-making.

### Supplementary Information

Below is the link to the electronic supplementary material.

Supplementary file1 (DOCX 16 KB)

## Data Availability

The data underlying this article will be shared on reasonable request to the corresponding author.
