# Supplementary material for: Determinants of health status in older patients with transthyretin cardiac amyloidosis: a prospective cohort study
Source: Aging Clin Exp Res. 2024 Apr 10;36(1):89. doi: 10.1007/s40520-024-02750-6 (PMC11006758; doi:10.1007/s40520-024-02750-6)
Supplement: Supplementary file 1 — Supplementary file1 (DOCX 16 KB) [file 40520_2024_2750_MOESM1_ESM.docx]

| **Supplementary Table 1. Univariable linear regression analysis to study factors associated with KCCQ.** | | | | | | | |
| --- | --- | --- | --- | --- | --- | --- | --- |
| **Variables** | **Unstandardized Coefficients** | | **Standardized Coefficients** | **t** | **p-value** | **95.0% Confidence Interval for B** | |
|  | **β** | **Std. Error** | **β** |  |  | **Lower Bound** | **Upper Bound** |
| **ATTR Type (v- vs wt-)** | 7.447 | 4.754 | 0.150 | 1.566 | 0.120 | -1.979 | 16.873 |
| **LVEF (per Δ %increase)** | 6.233 | 17.418 | 0.033 | 0.358 | 0.721 | -28.256 | 40.722 |
| **NAC Score (per Δ in class)** | -8.473 | 2.431 | -0.323 | -3.486 | 0.001 | -13.294 | -3.653 |
| **Age at diagnosis (per Δ year)** | -0.800 | 0.220 | -0.333 | -3.638 | 0.000 | -1.236 | -0.364 |
| **NYHA Class** | -13.235 | 2.809 | -0.418 | -4.712 | 0.000 | -18.805 | -7.665 |
| **Disease Duration (per Δ month)** | -0.334 | 0.080 | -0.384 | -4.156 | 0.000 | -0.493 | -0.175 |
| **Living Alone** | 10.112 | 4.891 | 0.337 | 1.965 | 0.020 | 2.801 | 19.338 |
| **Widower** | -6.939 | 4.478 | -0.149 | -1.550 | 0.098 | -15.818 | 1.939 |
| **mFI (per Δ increase)** | -48.765 | 10.908 | -0.403 | -4.471 | 0.000 | -70.398 | -27.131 |
| **ATTR**: Transthyretin cardiac amyloidosis; **LVEF**: Left Ventricular Ejection Fraction; **KCCQ**: Kansas City Cardiomyopathy Questionnaire; **mFI**: modified Frailty Index; **NAC**: National Amyloidosis Centre. | | | | | | | |
